# Surgical site infection and its associated factors following cesarean section: a cross sectional study from a public hospital in Ethiopia

**DOI:** 10.1186/s13037-017-0131-3

**Published:** 2017-06-12

**Authors:** Kelemu Abebe Gelaw, Amlaku Mulat Aweke, Feleke Hailemichael Astawesegn, Birhanu Wondimeneh Demissie, Liknaw Bewket Zeleke

**Affiliations:** 1 Department of Midwifery, College of Health Science and Medicine, Wolaita Sodo University, Wolaita Sodo, Ethiopia; 2 Department of Midwifery, College of Medicine and Health Sciences, Bahir Dar University, Bahir Dar, Ethiopia; 3 School of Public Health, College of Health Science and Medicine, Wolaita Sodo University, Wolaita Sodo, Ethiopia; 4 Department of Nursing, College of Health Science and Medicine, Wolaita Sodo University, Wolaita Sodo, Ethiopia

**Keywords:** Surgical site infection, cesarean section

## Abstract

**Background:**

A cesarean section is a surgical procedure in which incisions are made through a woman's abdomen and uterus to deliver her baby. Surgical site infections are a common surgical complication among patients delivered with cesarean section. Further it caused to increase maternal morbidity, stay of hospital and the cost of treatment.

**Methods:**

Hospital based cross-sectional study was conducted to assess the magnitude of surgical site infection following cesarean Site Infections and its associated factors at Lemlem Karl hospital July 1, 2013 to June 30, 2016. Retrospective card review was done on 384 women who gave birth via cesarean section at Lemlem Karl hospital from July 1, 2013 to June 30, 2016. Systematic sampling technique was used to select patient medical cards. The data were entered by Epi info version 7.2 then analyzed using Statistical Package for Social Sciences windows version 20. Both bivariate and multivariate logistic regression was done to test association between predictors and dependent variables. *P* value of < 0.05 was considered to declare the presence of statistically significantly association.

**Results:**

Among 384 women who performed cesarean section, the magnitude of surgical site infection following cesarean section Infection was 6.8%. The identified independent risk factors for surgical site infections were the duration of labor AOR=3.48; 95%CI (1.25, 9.68), rupture of membrane prior to cesarean section AOR=3.678; 95%CI (1.13, 11.96) and the abdominal midline incision (AOR=5.733; 95%CI (2.05, 16.00).

**Conclusions:**

The magnitude of surgical site infection following cesarean section is low compare to other previous studies. The independent associated factors for surgical site infection after cesarean section in this study: Membranes rupture prior to cesarean section, duration of labor and sub umbilical abdominal incision. In addition to ensuring sterile environment and aseptic surgeries, use of WHO surgical safety checklist would appear to be a very important intervention to reduce surgical site infections.

## Background

A cesarean section is a surgical procedure in which incisions are made through a woman's abdomen and uterus to deliver her baby. Cesarean section (C-section) may be necessary when vaginal delivery might pose a risk to the mother or baby when there is prolonged labor, fetal distress, or when the baby is presenting in an abnormal position. However, cesarean section can cause significant complications, disability or death, particularly in settings that lack the facilities to conduct safe surgeries or treat potential complications [1]. Since 1985, the international healthcare community has considered the ideal rate for cesarean section to be between 10% and 15%. Since then, cesarean sections have become increasingly common in both developed and developing countries. Due to this the worldwide continuous rise in the incidence of cesarean sections, the number of women with postpartum infection is expected to increase [1].

Pregnant women are at risk of infection during labor and delivery. Among surgical patients in obstetrics; Surgical Site Infections (SSIs) are the most common nosocomial infections, accounting for 38% of hospital acquired infections [2].

Although C-sections are performed in a sterile environment, the risk of surgical site infection always exists. Use of prophylactic antibiotics has been shown to significantly reduce post-cesarean infectious morbidity. The latest American College of Obstetrician and Gynecologist (ACOG)committee opinion recommends administration of antibiotics within 60 min of cesarean section and that where this is not possible the antibiotics be administered as soon as possible [3].

Most cesarean sections heal uneventfully within a predictable timeframe. However, for a small proportion of patients, the wound will develop complications. As a result, Surgical site infections are the most common post-operative complications even in hospitals with most modern facilities and standard protocols of preoperative preparation and antibiotic prophylaxis [4].

The average expected surgical site infections rate being 6–27% after C-section. These rates are increased in the presence of other risk factors such as gross contamination of the operative site, prolonged and premature rupture of membranes, obstructed labor, prolonged operative time, emergency operations, altered immune status, which are common in resource poor countries [5].

Risk of surgical site infection in developing countries is more than the developed countries (especially in sub Saharan Africa, the average wound infection rates are 2 or 3 times higher than developed countries) due to malnutrition, anemia, poverty and environmental pollution; poor preoperative preparation, wound contamination, poor antibiotic selection, or the inability of an immune-compromised patient to fight against the infection. These are avoidable in most circumstances by altering host, microbial and environmental factors in favor of the host [6].

cesarean sections carries a risk of infection 5 to 20 times that of normal delivery. It is the single most important risk factor for postpartum maternal infection which account for approximately 10% of pregnancy-related mortality. Contamination of the wound is present to some extent in all incisions thus adding significant morbidity and mortality [7].

Most of (15 to 80%) of post cesarean section infections may actually occur after initial discharge from the hospital. As a result, it is stressful for women from low income settings who develop a surgical site infection. Because these women often have very little practical experience on wound management and have to cope on their own at home [8].

Recovery from cesarean section is more difficult for women who develop postoperative infection. These infections may affect the pelvic organs, the surgical Wound and the respiratory and urinary tracts [9]. Patients with surgical site infections have a 2–11 times higher risk of death than those without surgical site infections and 77% of deaths associated with surgical site infections are directly related to the surgical site infection. Further it caused to increase stay of hospital and the cost of treatment [10]. Post cesarean Wound Infection is not only a leading cause of prolonged hospital stay but a major cause of the widespread aversion to cesarean delivery in developing countries [11].

Surgical site infection surveillance with feedback of surgical infection rates to surgeons is one of the successful strategies to help reduce surgical site infection. All hospitals with surgical services are recommended to undertake surveillance of surgical site infection [12]. Surveillance is common in high-income countries for a wide-range of surgical procedures but in sub-Saharan Africa including Ethiopia very few studies have been done on surgical site infections surveillance systems. In Ethiopia, surgical site infection after cesarean section still constitutes a problem. This can be explained for many reasons as many lack of budget, poor hygiene and nutrition, untrained staff, poor hand washing practice.

Therefore, proper assessment of magnitude and risk factors that predispose to surgical site infection after cesarean section is essential for developing targeted interventions to reduce its occurrence and complications. And it also helps in reducing hospital costs and length of patient stay in the hospital.

## Methods

### Study settings

The study was conducted at Lemlem Karl hospital which is a general hospital in Maichew, Tigiray region from July1, 2016 to August 30, 2016. The hospital is located within the city of Maichew away from 665 km Addis Ababa. According to 2007 Ethiopian central stastical agency report, the total populations of Maichew were 23,419. Of this 11, 204 were males and 13,395 were females. In this hospital l9, 200 patients visited per year. It awarded from Ethiopian hospital quality assurance in good documentation, clean and green hospitals in 2016. There are one hospital and two health centers. The Gynecology and Obstetrics department had 3 l beds. There were 1819 deliveries per year and 24 average cesarean sections per month. There were 9 midwives (5 diploma and 4 B.Sc), one Obstetrician and one emergency surgery surgeon.

### Study design

Hospital based cross sectional study design was conducted using retrospective chart review.

### Source population

The source population was all charts of women who gave birth via cesarean section at Lemlem Karl hospital.

### Study population

Study populations included in the study was selected charts of women who gave birth via cesarean section at Lemlem Karl hospital from July 1, 2013 to June 30, 2016.

### Eligibility criteria

Inclusion criteria

- ✓ All cards of women who underwent cesarean Delivery during the study period and having a diagnosis of SSI within 30 days of cesarean section at Lemlem Karl hospital.

### Sample size determination

The sample size was determined by using a single population proportion formula. The following assumptions were applied: p, prevalence of 50%(since there is no local data), d is the expected margin of error (5%), Z, the standard score corresponding to a 95% confidence interval and α, the risk of rejecting the null hypothesis (0.05). Accordingly the required sample size became 384.

### Sampling technique

Total of 847 women who have gave birth via cesarean section were recognized over three years at Lemlem Karl hospital from July 1, 2013 to June 30, 2016. From them using systematic sampling technique in every two interval 384 patient cards were identified and traced using registration number.

### Data collection instrument/process

Data were collected by using pretested checklist from July1, 2016 to August 30, 2016. All the variables of interest were assessed accordingly and the checklist was prepared in English. Two data collectors, who have diploma in midwife, were participated in the data collection process. Orientation was given to the data collectors on how to conduct the data collection. Using card number of patients, data collectors traced and collected data from randomly identified charts of cesarean section cases using checklist.

### Data analysis

The data were check for completeness, inconsistencies, and missing values and then coded, entered using EPI- info version 7.2. Then cleaned and analyzed using SPSS version 20. Descriptive statistics were computed to determine frequencies and summary statistics (mean, standard deviation, and percentage) to describe the study population in relation to socio-demographic and other relevant variables. Data were presented using tables, graphs and figures. Variables with P value <0.25 in bivariate analysis were transferred to multivariate analysis. Multiple logistic regressions were done to test the presence of association between predictors and dependent variables. *P* value ≤ 0.05, at 95% confidence interval was considered as cut point to declare the presence of statistically significant association.

### Data quality control

Orientation and appropriate supervision were done to data collectors by supervision made by the principal investigators. And completeness and consistency were checked every day during data collection. Checked was done on 10% of the total sample size in the same hospital on previous records. Appropriate modifications were made after analyzing the pre-test result before the actual data collection.

### Operational definitions

- Post cesarean section Surgical Site Infection: An infection which is developed after cesarean delivery on the operational site which is diagnosed by clinician.
- Prolonged hospital stay; defined as hospital stay lasting more than 7 days.
- Prolonged operation time; defined as cesarean section lasting more than one hour from skin incision to last skin stitch.

## Results

### Socio demographic Characteristics of women underwent cesarean section at Lemlem Karl hospital 2013–2016

Among the total 384 mothers included for the study operated for delivery during the study period in Lemlem Karl hospital. The mean (±SD) of the mothers’ age was 27(±5) years. The minimum and maximum age in years were 16 and 43. Majority of mothers age ranged from 20–34(89.3%) and ≥35 (6.9%). 228(59.4%) were come from rural area, 347(90.4%) Tigiray, and 334 (87%) were orthodox (Table 1).

**Table 1:** Shows distribution of Socio demographic Characteristics of women underwent cesarean section from July1, 2013- June 30,2016 at LKH (*n* = 384)

Variables		Frequency(*n*)	Percentage (%)
Age	<_19	15	3.9
	20–34	343	89.3
	>_35	26	6.8
Address	Urban	156	40.6
	Rural	228	59.4
Region	Tigre	347	90.4
	Amhara	18	4.7
	Afar	19	4.9
Religion	Orthodox	334	87
	Muslim	39	10.1
	Protestant	10	2.6
	Catholic	1	0.3

### Obstetrics, medical and operation characteristics of women underwent cesarean section in Lemlem Karl hospital 2013–2016

Majority of mothers (86.5%) were Para 1 up to 4 and 52 (13.5%) was Multipara. Almost all mothers (98.4%) had antenatal care follow-up, only two mothers had gestational diabetes mellitus. Fourteen (3.6%) mothers were developed pregnancy induced hypertension; there was one mother who had cardiac diseases. Seven (1.8%) case were HIV reactive and 6 of them on Antiretroviral therapy.

Most mothers operated at term pregnancy 303 (78%), 326(84.9%) were emergency cesarean section, 306(93.9%) operation were done with on labor. among mothers who was on labor before operation, 64(21.1%) were stayed more than 24 h. Meanwhile157 (51%) mothers were membrane ruptured prior to cesarean section. Most of mothers356 (92.7%) were operated thirty up to sixty minutes and mean operation was 56 min.

Of 384 studied participant, 33(8.6%) had history of cesarean section and 304(79.2%) were given spinal anesthesia. Most of cesarean section did by junior students191 (40.7%). Almost all (99%) mothers were taken antibiotics prophylaxis. On post operation hematocrit determination, 329(85.7%) were greater than thirty and 55(14.3%) were less than equal to thirty. The most common abdominal incision were Pfanestile281 (73.2%) (See Table 2).

**Table 2:** Shows the distribution of obstetrics, medical and operation characteristics of women underwent cesarean section from July 1, 2013- June 30, 2016 at LKH (*n* = 384)

Variables		Frequency (*n*)	Percentage (%)
Parity	1–4	332	86.5
	≥5	52	13.5
ANC follow up	Yes	378	98.4
	No	6	1.6
Diabetes Mellitus	Yes	2	0.5
	No	382	99.5
PIH	Yes	14	3.6
	No	370	96.4
Cardiac diseases	Yes	1	0.3
	No	383	99.7
HIV status	Reactive	7	1.82
	None reactive	357	92.97
	Unknown	20	5.21
On Antiretroviral therapy (ART)	Yes	6	85.7
	No	1	14.5
Gestational age	<37 weeks	45	11.7
	37-42wks	303	78.9
	>42 wks.	36	9.4
Emergency C/S	Yes	326	84.9
	No	58	15.1
Labor status	Yes	306	93.9
	No	20	6.1
Duration of labor	<24 hours	240	78.9
	≥24 hours	64	21.1
Membrane rupture before c/s	Yes	157	51
	No	151	49
Duration of membrane rupture	<12 hours	115	73.2
	≥12 hours	42	26.8
Duration of C/S	30–60 min	356	92.7
	>60 min	28	7.3
History of C/S	Yes	33	8.6
	No	351	91.4
Anesthesia used	General anesthesia	80	20.8
	Spinal anesthesia	304	79.2
C/S performed by	Obstetrician	43	11.2
	MSc student	341	88.8
Antibiotics prophylaxis’s	Yes	380	99
	No	4	1
Post-operative hematocrit	≤30	55	14.3
	>30	329	85.7
Types of abdominal incision	Pfannenstiel	281	73.2
	Midline	103	26.8

❖ IESO-integrated emergency surgery and obstetrics

❖ MSC-Master of Science in clinical midwifery

### Magnitude of surgical site infection following cesarean section in Lemlem Karl hospital 2013–2016

**Fig. 1:**
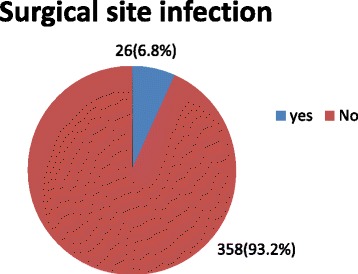
Magnitude of surgical site infection at Lemlem Karl hospital, Maichew Tigray, 2013–2016

The Magnitude of surgical site infection following to cesarean section was 26(6.8%). For twenty cases of women the SSI were detected before discharged and six cases were after discharged. Fourteen (53.8%) case stayed at hospital more than seven days (see Fig. 1).

### Factors associated with surgical site infection following cesarean section

There were five variables in binary logistic regression which had p value of ≤ 0.25; and became candidate for multiple logistic regression; duration of labor, rupture of membrane before cesarean section, types of anesthesia, post-operative hematocrit and types of abdominal incision.

In the multiple logistic analysis; duration of labor, rupture of membrane prior to cesarean section, and types of abdominal incision were significantly associated (*p* < 0.05). Mothers who were on labor for more than 24 h before cesarean section were 3.48 times more likely risk for surgical site infections than those less than 24 h AOR=3.48; 95%Cl (1.25, 9.68). The chance of developing surgical site infections that had rupture of membrane before cesarean section was 3.68 times more likely than intacted membrane (AOR=3.678; 95%Cl (1.13, 11.96). Mothers who had midline abdominal incision were 5.73 times more likely to develop surgical site infections as compare with pfannenstiel abdominal incision AOR=5.733; 95%Cl (2.05, 16.00) (see Table 3).

**Table 3:** Shows the bivariate and multivariate association of surgical site infections and independent factors mothers underwent cesarean section at LKH July 1, 2013-July 30, 2016 (*n* = 384)

Variables		Surgical site infection		COR (95%Cl)	AOR (95%Cl)
		No	Yes		
Duration of labor	<24 hrs	231 (80.8%)	9 (50%)	1	1
	≥24 hrs	55 (19.2%)	9 (50%)	4.2 (1.59–11.08)	3.48 (1.25–9.68**)
Rupture of membrane prior to C/S	No	147 (50.7%)	4 (22.2%)	1	1
	Yes	143 (49.3%)	14 (77.8%)	3.598 (1.16–11.19)	3.678 (1.13–11.96**)
Types of anesthesia	Spinal	289 (80.7%)	15 (57.7%)	1	1
	General	69 (19.3%)	11 (42.3%)	3.071 (1.35–6.98)	2.127 (0.66–6.83)
Post operation hematocrit	>30	312 (82.2%)	17 (65.4%)	1	1
	≤30	46 (12.8%)	9 (34.6%)	3.59 (1.51–8.53)	2.435 (0.67–8.85)
Types of abdominal incision	Pfanestile	271 (75.7%)	10 (38.5%)	1	1
	Midline	87 (24.3%)	16 (61.5%)	4.984 (2.18–11.39)	5.733 (2.05–16.00**)

** *P*- < 0.05 statically significant

## Discussion

The prevalence of surgical site infection following cesarean section in this study was 6.8%. This finding was lower than that found in Jimma referral hospital 11.4%; however included all obstetrical procedures (cesarean sections, abdominal hysterectomies and Destructive Vaginal Deliveries), unlike this study was focus on cesarean sections [13]. The finding was again lower than the value obtained from limbe, Cameroon which was 19.4%. The discrepancy in the results could be due to the fact that it was included both surgical and maternity wards [14].

This study approximately similar to others study done in three selected sub Saharan country (Democratic Republic of Congo, Burundi and Leone) which was 7.3% and Guwahati, Assam which was 6.03%[15, 16].

This finding much lower than finding on Zimbabwe which was (29%). This difference might be it was cohort prospective study and was done in two big referral teaching hospitals in the countries where as this research was done general hospital. Overcrowding in the wards is a precursor of infection [17].

This study is also low compared to study done in Kenyatta hospital, Kenya which was 22%. This is probably due to the time of prophylaxis antibiotics were given before skin incision in our set up but in Kenyatta hospital 97.2% were given after operation. Giving prophylactic antibiotics before cesarean section has been a normal step for cesarean section surgeries as it obviously decreases morbidity rate of maternal infections after operation especially when compared to giving antimicrobials after umbilical cord clamping. Interestingly, giving prophylactic antibiotics before cesarean delivery has no major effects on mothers or newborn babies. Study in Kenya had post discharge surveillance method of monitoring SSI after hospital discharge [18].

On the other hand this prevalence is higher than research done in Guangdong, china (0.7%); Oman, Saudi Arabia (2.66%); the possible reason may be; be due in the study area(china)it was account for almost 70% of births in some urban areas but in this study majority were rural areas(low socioeconomic status) [12, 19].

Prolonged labor was noted to be an independent risk factor for surgical site infection in this study. Women with labor duration greater than 24 h had 3.5 times more likely developing post cesarean wound infection (AOR=3.48; 95%Cl(1.25,9.68). It is further supported by other studies in Cambodia. This could be attributed to as duration of labor increases, the number of vaginal examinations also increase and repeated vaginal examinations increase the chance of iatrogenic contamination during examination [11].

This study also indicated that significant association was noted between rupture of membrane prior to cesarean section and surgical site infections. Mothers with ruptured membrane prior to cesarean section were 3.7 times to more likely to have surgical site infection those mothers had no rupture of membrane (AOR=3.69; 95%Cl(1.13,11.96). This is in line to study done Dhulikhel hospital Kathmandu University, Nepal. This might be; when the membranes rupture, the amniotic fluid is no longer sterile and this may act as a transport medium by which bacteria may come into contact with the uterine and skin incisions thereby leading to risk of developing surgical site infections [20].

The size of incision is matter for infection in this study also mothers who had midline abdominal incision 5.7 times more likely to develop surgical site infections as compare to pfannenstiel incision (AOR=5.733; 95%Cl(2.05,16.00).it is in line to finding in Nnewi, Nigeria [21].

## Conclusions

The result obtained for the surgical site infection following cesarean section is lower than other previous studies from developing countries but it is higher than studies done in developed countries. Independent risk factors were identified for increased risk of surgical site infection on this study, Such as, prolonged labor, rupture of membrane before cesarean section and types of abdominal incision. Therefore to reduce surgical site infection the hospital infection control system as well as surgical site infection surveillance program has to be established. In addition, sterile environment and aseptic surgeries, use of WHO surgical safety checklist would appear to be a very important intervention to reduce surgical site infections.
